# Floating-dislocated elbow in adults: Case reports and literature review

**DOI:** 10.1097/MD.0000000000030891

**Published:** 2022-09-30

**Authors:** Bogdan Veliceasa, Mihaela Pertea, Dragos Popescu, Claudiu Adrian Carp, Roxana Pinzaru, Bogdan Huzum, Ovidiu Alexa, Cristina Strobescu-Ciobanu, Alexandru Filip

**Affiliations:** a University of Medicine and Pharmacy “Grigore T. Popa” Iasi, Romania; b Department of Orthopaedics and Traumatology, “Sf. Spiridon” Emergency Hospital Iasi, Romania; c Department of Plastic Surgery and Reconstructive Microsurgery, “Sf. Spiridon” Emergency Hospital Iasi, Romania; d Department of Vascular Surgery, “Sf. Spiridon” Emergency Hospital Iasi, Romania.

**Keywords:** dislocated, elbow, floating, fracture

## Abstract

Floating-dislocated elbow is a severe and extremely rare injury in adults. Reviewing the literature, we found around 6 case reports regarding floating-dislocated elbow in adults.

**Patient concerns and diagnoses:**

We report 2 cases of this unusual injury association. Both patients suffered a high energy trauma – fall from a height. Initial X-rays (radiography) revealed in both cases the fractures above and below the elbow (floating elbow) and associated elbow dislocation (floating-dislocated elbow). One case was a type IIIB Gustilo-Anderson open fracture-dislocation with an intra-articular component (olecranon fracture).

**Interventions and outcomes:**

Each case had his own management problem regarding what to treat first: the dislocation or the associated fractures? Fractures were treated surgically by reduction and internal fixation, and after elbow dislocation reduction, the upper limb was immobilized in a long, well-padded plaster, with the elbow in 90° of flexion, for 3 weeks. Bone union was observed radiographically at 2 months after surgery in both cases. At the 2-year follow-up we recorded full upper limb recovery in terms of muscular trophism and elbow full range of motion.

**Lessons:**

In addition to adding 2 new cases to a lower number of such lesion associations in adults, we also added a new variant of floating-dislocated elbow which has not been reported until now in the literature. Prompt management of these injuries, with stable fixation of the fractures allowed for early rehabilitation with excellent 2-years functional outcome.

## 1. Introduction

The term “floating” is used in orthopedic trauma to describe an injury pattern characterized by the fractures of the bones above and below of a joint. Until now there are 12 different injuries that are described as floating.

The term “floating elbow” was first introduced by Stanitski and Micheli in 1980 to describe an unusual injury pattern in children in which a humeral and forearm (radius and/or ulna) fracture disconnects the elbow from the remaining limb. Later, Rogers et al, in 1984, introduced the same term to define the equivalent injury in adults.^[1,2]^ Although relatively common in children, this injury pattern is rare in adults.

A rarer associated injury in adults is the “floating-dislocated elbow” which associates fractures above and below the elbow (floating elbow) with elbow dislocation. Reviewing the literature, we found around 6 case reports regarding floating-dislocated elbow in adults.^[2–7]^

They are severe injuries and surgical fixation is required.^[8]^ Early recognition and prompt management could lead to better prognosis. These injuries are severe and require surgical fixation.^[8]^ Early recognition and prompt management may improve prognosis. Being associated with many complications such as infection, nonunion, and nerves injuries, even with prompt surgical treatment the outcome is uncertain.^[9]^

## 2. Methods

We report 2 cases of floating dislocated elbow describing the clinical findings, radiological appearances, and management challenges. Both previously mentioned patients gave their consent to participate in this study and authorized the photographs for publishing. The approval of the Ethical Boards of the Emergency Hospital “Sf. Spiridon” Iasi was obtained according to international regulations.

### 2.1. Case 1

A 64 years old female was attended at the emergency room after a fall from a height of 3 m. From the patient history we found out that she felt from a ladder on her left palm with the elbow extended and arm in approximately 90° of abduction and landed on her left side. Physical examination revealed markedly swollen upper limb with deformity, with no cutaneous and neurovascular damage.

**Figure 1.**
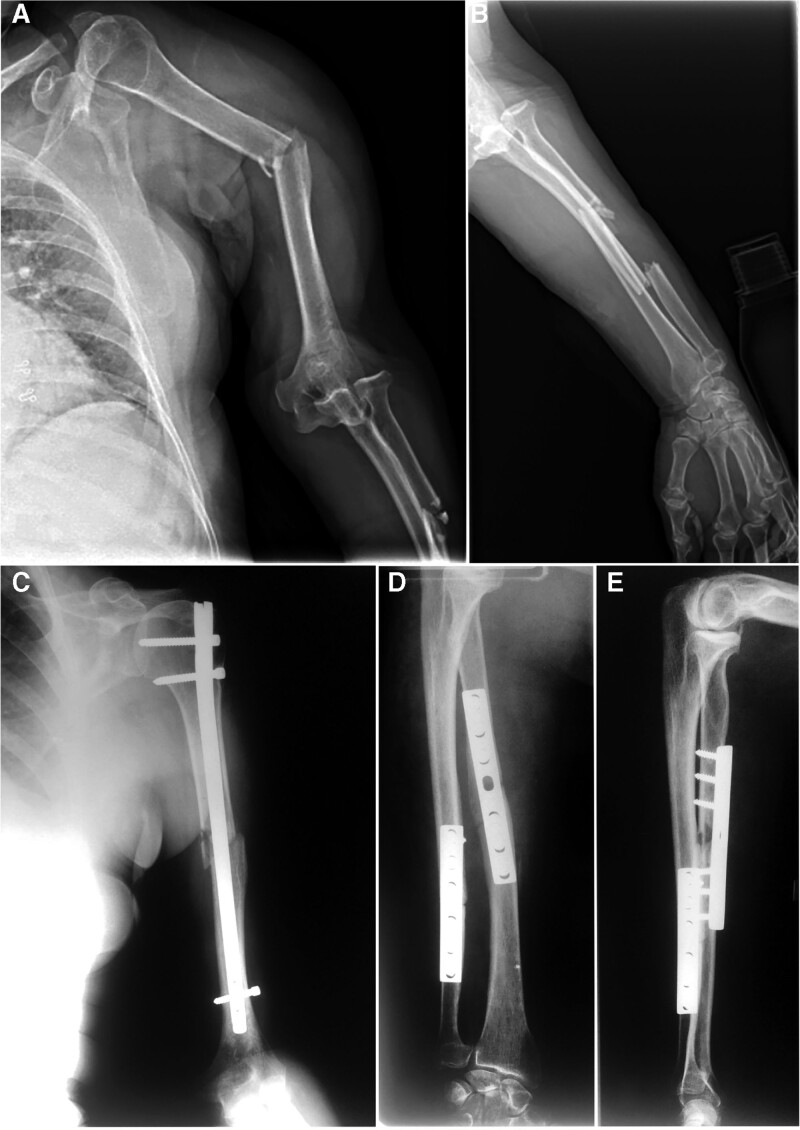
(A) Initial X-ray (antero-posterior view) of the arm – displaced middle third humeral shaft fracture and postero-lateral elbow dislocation. (B) Initial X-ray (lateral view) of the forearm – proximal third radial shaft and distal third ulnar fractures, and posterior elbow dislocation. (C) Postoperative X-rays (antero-posterior view) of the arm – humeral shaft fracture reduction and fixation with a locked intramedullary nail. (D, E) Postoperative X-rays (antero-posterior and lateral views) of the forearm – reduction of fractures and fixation with plates, reduction of the elbow dislocation.

Initial radiography (X-rays) showed a short oblique fracture of the midshaft of the humerus, a postero-lateral dislocation of the elbow and midshaft fracture of the forearm bones (Fig. 1A and B).

The patient underwent reduction of the elbow dislocation followed by closed reduction and internal fixation (CRIF) of the humeral shaft fracture with an intramedullary nail and open reduction and internal fixation (ORIF) with plates of the forearm fractures (Fig. 1C–E).

### 2.2. Case 2

**Figure 2.**
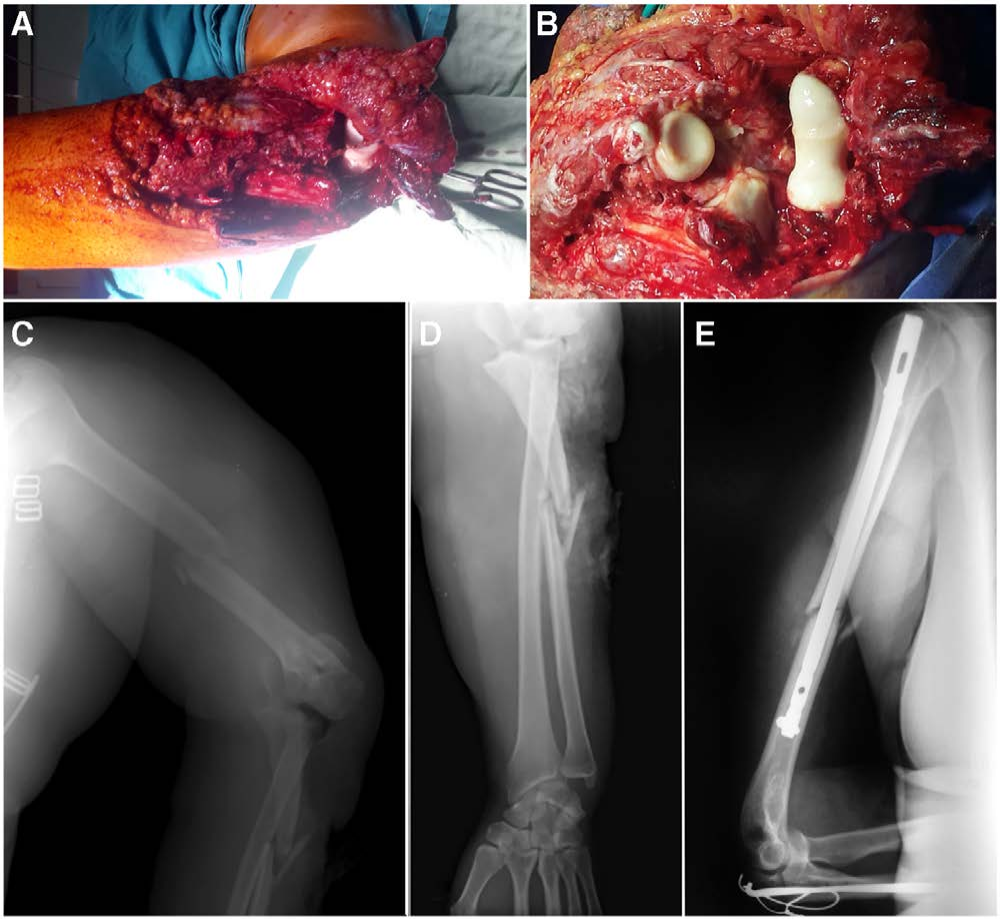
(A) Clinical appearance of the posttraumatic elbow wound. (B) Intra-articular elbow wound detail with direct visualization of elbow dislocation and olecranon fracture. (C) Initial X-ray (antero-posterior view) of the arm – displaced middle third, oblique, humeral shaft fracture, olecranon fracture and postero-medial elbow dislocation. (D) Initial X-ray (antero-posterior view) of the forearm – displaced proximal third, complex ulnar fracture, also reveals the medial elbow dislocation. (E) Postoperative X-ray (lateral view) of the arm - humeral shaft fracture reduction and fixation with a locked intramedullary nail, reduction of the olecranon and proximal third ulnar shaft fractures and fixation with a tension band, reduction of the elbow dislocation.

A 49 years old female was attended at the emergency room after a road traffic accident. She declared that she fell to the ground from a height of 2 m (from a horse-drawn cart laden with hay) and landed on her left palm with the forearm supine, elbow fully extended and the shoulder in abduction and retropulsion. Physical examination revealed a markedly swollen upper left limb with deformity. Also, we noted an extended intra-articular wound, 10 cm in width and approximately 15 cm in length, at the level of the elbow and proximal third of the forearm, with the detachment of a fascio-cutaneous flap with distal pedicle (Fig. 2A and B). It was classified as type IIIB Gustilo-Anderson open fracture-dislocation.^[10]^ Initial X-rays revealed: midshaft humeral fracture, olecranon fracture, complex midshaft ulna fracture, and postero-medial dislocation of the ulnar-humeral joint (Fig. 2C and D).

The patient underwent emergent surgery: in the first instance, debridement with excision of devitalized tissues and anchoring the fascio-cutaneous flap with a few stitches; in the second instance, ORIF of the olecranon fracture with a tension band and CRIF of the ulnar shaft fracture with Kirshner wire (we used long Kirshner wires for the fixation of the olecranon which were advanced into the ulnar medullary canal to fix the ulnar shaft fracture at the same time) were performed. Elbow dislocation was reduced concomitantly with olecranon fracture reduction. Due to the risk of infection, the surgical treatment for the humeral shaft fracture was delayed.

The upper limb was immobilized in a posterior, above the elbow, plaster and checked every day to rule out any signs of infection. Antibiotic therapy was started for the prophylaxis of infections according to the hospital protocol. Seven days later, in the absence of any sign of local infection, we decided to surgically treat the humeral shaft fracture by CRIF with an intramedullary nail (Fig. 2E).

In both patients, after surgery the arm was immobilized with elbow flexed at 90° using a long, well-padded plaster for 3 weeks. Then, the patients were encouraged to begin upper limb rehabilitation with joint range of motions and muscle strengthening exercises.

Bone union was seen on radiographs 2 months postoperatively in both cases.

At the 2-year follow-up we noticed full upper limb recovery in terms of muscle trophism and full elbow flexion/extension and pronation/supination range of motion (Table 1). According to the Mayo elbow performance score, the functional outcome was excellent in both cases.^[11]^

**Table 1:** Patients values of elbow range of motion at the 2-year follow-up.

	Flexion	Extension	Pronation	Supination	Mayo elbow performance score
Case 1	145°	0°	65°	76°	100 pts
Case 2	130°	−5°	60°	72°	95 pts

## 3. Discussion

Unusual fracture-dislocation associations were reported over the time in the literature.^[12]^ Of these, floating elbow is a rare injury pattern in adults characterized by ipsilateral diaphyseal fractures of the humerus, radius and ulna. In even fewer cases, this injury could be associated with elbow dislocation in patients sustaining high-energy traumatic injuries.

The spectrum of injury can vary greatly, in direct relationship with the force dissipated.^[13]^ Intra-articular and shaft fractures with associated elbow dislocation that can functionally act as floating elbow have been described previously in case reports. These are called “variants”, and therefore there is no universal classification.^[5–7]^

Based on their case report and the 3 cases reported before 2006, De Carli et al were the first to propose a classification into 3 types.^[5]^ Later on, in 2017, El Ayoubi et al described a new variant that was not included in that classification: association of proximal humeral fracture with posterolateral elbow dislocation and distal radius fracture which made the whole upper limb unstable.^[6]^ Of our 2 cases, case 1 could be classified as De Carli type I. As to our case 2, we could not find a similar report in the English literature, more complex due to the severity of injury both to the soft tissue and articular component. Thus, 2 more types should be added to the initial De Carli classification: type IV – the case reported by El Ayoubi et al, and type V – our case 2 (Table 2).^[6]^

**Table 2:** Variants of floating-dislocated elbow.

Variants	Description	Cases No.	Authors/years
Type I	Floating-dislocated elbow without articular bone injury	1	Rogers JF et al,^[2]^ 1984
		1	Viegas SF et al,^[3]^ 1989
		1	**Our case 1**
Type II	Floating-dislocated elbow with distal radio-ulnar joint dislocation	1	Sarup S et al,^[4]^ 1997
Type III	Floating-dislocated elbow with articular fracture of the distal aspect of the humerus	1	De Carli P et al,^[5]^2006
Type IV	Floating-dislocated elbow with distal radius fracture	1	El Ayoubi A et al,^[6]^ 2017
Type V	Floating-dislocated elbow with articular fracture of the olecranon	1	**Our case 2**

The mechanism of trauma that resulted in this associated injury is quite difficult to determine based on patient history. Posterior elbow dislocations typically occur from a fall on an outstretched hand, and an elbow loaded axially in a varus/valgus position, with the forearm supinated/pronated can result in lateral/medial variants. The remaining forces further cause fractures above and below the elbow. The injury mechanisms in the cases reported in the literature were: the winch system of a shrimp boat, car accident in 1 case, and in other 2 cases fall from a height of 4 m, without any other details.^[3,5–7]^ In our 2 cases, the mechanisms involved could be partially described based on patient history: fall from height landing on the palm with fully extended elbow, and the shoulder abducted in in case 1 and partially in abduction and in retropulsion in case 2.

Several treatment options for the floating elbow have been suggested, mainly related to the humeral fracture: closed reduction and cast immobilization, ORIF, external fixation.^[2,7–9]^ Because Rogers et al reported a high rate of humeral nonunion in the absence of stable fixation, Simpson and Jupiter and Yokoyama et al recommended the stable internal fixation of the fractures in floating elbow injuries.^[2,8,14]^ In our cases the treatment consisted in emergency reduction of elbow dislocation followed by internal fixation of both fractures, above and below the elbow. This allowed us early reeducation with excellent functional outcome.

Another issue is what lesion should be addressed first. In our case 1, the lesions were addressed in the following order: dislocation reduction, humeral fracture, and forearm fractures. In case 2, being in front of an type IIIB Gustilo-Anderson open fracture-dislocation, the order was: the wound; the olecranon and the ulnar shaft fracture that allowed a stable reduction of the dislocation, and the humeral shaft fracture.

## 4. Conclusions

Here we reported 2 cases of this unusual injury association of which 1 case of type IIIB open elbow dislocation with humeral shaft fracture, olecranon and upper third ulnar shaft fracture, a variant that we could not find a similar report in the English literature. Prompt management of these injuries, with stable fixation of the fractures allowed for early rehabilitation with excellent 2-years functional outcome.

## Author contributions

**Conceptualization:** Bogdan Veliceasa, Ovidiu Alexa, Alexandru Filip.

**Data curation:** Bogdan Veliceasa, Claudiu Adrian Carp, Bogdan Huzum, Alexandru Filip.

**Formal analysis:** Bogdan Veliceasa, Bogdan Huzum.

**Investigation:** Mihaela Pertea, Dragos Popescu, Bogdan Huzum, Cristina Strobescu-Ciobanu.

**Methodology:** Mihaela Pertea, Dragos Popescu, Claudiu Adrian Carp, Roxana Pinzaru, Cristina Strobescu-Ciobanu, Alexandru Filip.

**Project administration:** Claudiu Adrian Carp.

**Resources:** Claudiu Adrian Carp, Roxana Pinzaru, Cristina Strobescu-Ciobanu.

**Software:** Cristina Strobescu-Ciobanu.

**Supervision:** Mihaela Pertea, Dragos Popescu.

**Validation:** Dragos Popescu.

**Visualization:** Roxana Pinzaru, Ovidiu Alexa.

**Writing – original draft:** Bogdan Veliceasa, Dragos Popescu, Roxana Pinzaru, Ovidiu Alexa, Alexandru Filip.

**Writing – review & editing:** Bogdan Veliceasa, Ovidiu Alexa, Alexandru Filip
